# Hyoscine as a Sedative

**Published:** 1889-11-09

**Authors:** 


### HYOSCINE AS A SEDATIVE.

Dr. Walter S. Colman, registrar and pathologist to the hospital for the paralysed, and Dr. James Taylor, house physician to the hospital, contribute notes on the value of hyoscine.* The preparation employed was a 1 in 500 solution of the hydrohomata of hyoscine in distilled water, used as a subcutaneous injection, five minims of which is equal to a dose of $\frac{1}{100}$ gr. of the salt. Eight cases of mental excitement are recorded, in which more or less good effects resulted. On the other hand, two cases of other affections are given, illustrating the occasional unpleasant effects following the use of hyoscine. The principal unpleasant effects were restlessness, delusions, dryness of the mouth, dimness of sight, and delirium with double consciousness. From all brought forward by the observers it would appear that the real use of hyoscine is as a sedative on cases of mental excitement, and that it is not advisable to use the preparation for such maladies as epilepsy or paraplegia. Ringer states that hyoscine is especially useful in the more violent forms of intermittent mania when it is difficult to restrain the patients, and Drs. Coleman and Taylor's observations confirm this. The toxic symptoms produced by hyoscine are not unlike those arising from datura, which probably might be similarly useful.

